# ^18^F-FDG PET/CT-based deep learning radiomics predicts 5-years disease-free survival after failure to achieve pathologic complete response to neoadjuvant chemotherapy in breast cancer

**DOI:** 10.1186/s13550-023-01053-7

**Published:** 2023-12-06

**Authors:** Xingxing Zheng, Yuhong Huang, Yingyi Lin, Teng Zhu, Jiachen Zou, Shuxia Wang, Kun Wang

**Affiliations:** 1 Department of Breast Cancer, Cancer Center, Guangdong Provincial People’s Hospital (Guangdong Academy of Medical Sciences), Southern Medical University, Guangzhou, China; 2 Shantou University Medical College, Shantou, China; 3 Guangdong Medical University, Zhanjiang, China; 4 Department of Nuclear Medicine and PET Center, Guangdong Provincial People’s Hospital (Guangdong Academy of Medical Sciences), Southern Medical University, Guangzhou, China

**Keywords:** Breast cancer, Deep learning, Radiomics, PET/CT, Neoadjuvant chemotherapy

## Abstract

**Background:**

This study aimed to assess whether a combined model incorporating radiomic and depth features extracted from PET/CT can predict disease-free survival (DFS) in patients who failed to achieve pathologic complete response (pCR) after neoadjuvant chemotherapy.

**Results:**

This study retrospectively included one hundred and five non-pCR patients. After a median follow-up of 71 months, 15 and 7 patients experienced recurrence and death, respectively. The primary tumor volume underwent feature extraction, yielding a total of 3644 radiomic features and 4096 depth features. The modeling procedure employed Cox regression for feature selection and utilized Cox proportional-hazards models to make predictions on DFS. Time-dependent receiver operating characteristic (ROC) curves and the area under the ROC curve (AUC) were utilized to evaluate and compare the predictive performance of different models. 2 clinical features (RCB, cT), 4 radiomic features, and 7 depth features were significant predictors of DFS and were included to develop models. The integrated model incorporating RCB, cT, and radiomic and depth features extracted from PET/CT images exhibited the highest accuracy for predicting 5-year DFS in the training (AUC 0.943) and the validation cohort (AUC 0.938).

**Conclusion:**

The integrated model combining radiomic and depth features extracted from PET/CT images can accurately predict 5-year DFS in non-pCR patients. It can help identify patients with a high risk of recurrence and strengthen adjuvant therapy to improve survival.

**Supplementary Information:**

The online version contains supplementary material available at 10.1186/s13550-023-01053-7.

## Introduction

Breast cancer is the most commonly diagnosed cancer in women, posing a significant threat to their health and survival [1]. Locally advanced breast cancer is conventionally treated with neoadjuvant chemotherapy (NAC) [2]. Pathological complete response (pCR) after NAC is associated with improved disease-free survival (DFS) and overall survival (OS) outcomes [3–7]. For patients who do not achieve pCR, accurate prognostic assessment is crucial in determining appropriate treatment escalation or de-escalation, minimizing overtreatment for those with favorable prognosis and intensifying adjuvant therapy for those with an unfavorable prognosis to enhance survival. Known prognostic factors for breast cancer include age, axillary nodal status, tumor size, pathology, grade, and peritumoral lymphatic and vascular invasion [8]. Residual cancer burden (RCB) scores are commonly used as an independent predictor of survival. A higher RCB grade indicates a poorer prognosis [9]. The RCB scoring system focuses solely on the pathological factors related to tumors and lymph nodes after NAC. To enhance prognostic accuracy, the combination of multiple prognostic indicators has been advocated, as shown in recent studies [10].

The National Comprehensive Cancer Network (NCCN) Task Force recommends ^18^F-2-deoxy-2-fluoro-d-glucose (FDG) positron emission tomography/computed tomography (PET/CT) assessment for patients with locally advanced tumors that have higher risks of distant metastases due to nodal involvement [11]. PET/CT has distinct advantages over traditional imaging techniques because it provides information about the tumor’s metabolic and biological aspects characteristics and the patient's prognosis [12, 13]. Notably, PET texture analysis has been instrumental in providing critical prognostic information for solid tumors [14, 15].

Radiomics is a nascent area of research that transforms medical imaging features into quantifiable data used in decision support systems [16]. By improving knowledge of tumor behavior, radiomics holds promise to guide patient management at the bedside [17], thus bringing personalized medicine closer to reality [18]. Moreover, radiomics provides a comprehensive and non-invasive assessment of tumors, eliminating the need for invasive biopsies and reducing the potential for sampling errors [19]. Deep Learning (DL) is a method that directly extracts characteristics from images to produce faster and more precise results when compared to classical Machine Learning. DL models are capable of acquiring hierarchical characteristics from the image data through multiple layers, which operate in a depth-based manner for feature acquisition [20]. In recent research studies [21–23], DL has exhibited favorable performance concerning the detection and diagnosis of cancer. Nevertheless, the utilization of PET/CT radiomics and deep features for prognosis prediction in non-pCR breast cancer patients remains underexplored.

Hence, our study aimed to develop an integrated model, encompassing clinicopathologic factors, radiomic features, and depth information from baseline PET/CT scans. This combined model seeks to provide accurate long-term survival predictions for non-pCR patients, identifying high-risk populations and informing future treatment decisions.

## Methods and materials

### Patients

This retrospective study included 105 newly diagnosed stage II-III breast cancer patients who underwent PET/CT examination at Guangdong Provincial People's Hospital between January 2009 and December 2014. All patients received surgical treatment after completing NAC. The present study obtained approval from the Institutional Review Board of Guangdong Provincial People's Hospital. This study was performed following the Declaration of Helsinki's principles and requirements. As this was a retrospective study, formal consent was not obtained.

Eligible patients were female, aged 18 years and above, with histologically confirmed invasive breast cancer and clinically staged as T2-4N0-3M0 or T1cN1-3M0. Additionally, patients must have undergone PET/CT before receiving NAC and were confirmed as non-pCR after curative surgery following NAC completion. Patients who underwent direct surgical treatment, exhibited distant metastases, suffered from bilateral breast cancers, or had a previous history of breast cancer were excluded.

Tissue type, nuclear grading, hormone receptor expression, proliferative activity (Ki67), and human epidermal growth factor receptor 2 (HER2) status were recorded, and patients were classified based on St. Gallen molecular subtypes [24]. Immunohistochemical (IHC) markers for estrogen receptor (ER), progesterone receptor (PR), and HER2 were used to categorize patients into the following three molecular subtypes: luminal (ER-positive and/or PR-positive and HER2-negative), HER2-enriched (HER2-positive regardless of hormone receptor status) and triple-negative (ER-negative, PR-negative, and HER2-negative).

### Breast cancer diagnosis and neoadjuvant chemotherapy regimen

The IHC results were judged using specific criteria. ER and PR positivity were defined as tumor cell nuclei with ≥ 1%, while those with < 1% were considered negative. HER2-positivity was defined as HER2 (+++), whereas HER2 (−) and HER2 (+) were considered HER2-negative. HER2 (++) required examination using fluorescence in situ hybridization (FISH) for HER2 gene expansion to enhance its detection. Gene amplification determined HER2-positive status, otherwise, it was considered negative. IHC results and non-pCR status were determined by our pathologists with 10 years of work experience. All NAC regimens were administered according to NCCN recommendation guidelines. Patients with HER2-positivity also received HER2-targeted therapy. Surgical excision was performed 2–3 weeks after completing NAC.

### Follow-up evaluation

All patients were subject to postoperative follow-up starting from the day of their surgical intervention. During the five years following surgery, patients were examined every three to six months and had mammograms done once a year. Physical examinations and mammograms were conducted regularly on the patient for up to a decade following their surgical intervention. CT, PET/CT, or tissue biopsy was utilized to diagnose recurrence in cases where it was suspected. Recurrence was defined as any unambiguous evidence of the appearance of new cancer foci in a previously deemed disease-free patient. The duration of DFS was calculated by measuring the time from surgical intervention until the first evidence of cancer recurrence, death, or the latest clinical encounter confirming the absence of disease.

### PET/CT imaging

To prepare for the scan, all patients were required to fast for a minimum of 6 h. Blood glucose levels must be below 10.0 mol/L before injection and the FDG dose is 7.4 MBq /kg. After intravenous injection, the patient rests in a dark room for 60 min before undergoing a 3D PET/CT measurement (Biograph16, 120 keV, 50 mAs). The CT scan was performed initially, covering the area from the proximal thighs to the head. PET data were then acquired over the same extent, after the CT scan, at a rate of 2–3 min per bed position. The process of analyzing PET and CT images that had been co-registered was executed utilizing a specialized workstation.

### Image segmentation and preprocessing

The analysis and interpretation of the PET/CT images were performed by two experts in nuclear medicine. Using software (3D-Slicer), a large three-dimensional area of interest (3D-ROI) was plotted in each patient around the original breast lesion. The determination of ROI was done using a semi-automatic segmentation algorithm to ensure reproducibility and reliability. The open-source software 3DSlicer (https://www.slicer.org) is widely used for volume analysis of imaging data [25]. Then, the two expert doctors manually adjusted the ROI measurement to ensure measurement reliability. If there was a 5% difference between the two doctors, A senior nuclear medicine scientist would review and determine ROI. The details on image pre-processing are shown in the Additional file 1: S4.

### Feature extraction

In this study, 3644 radiomic features and 4096 depth features were extracted from PET/CT images and ROI. We used PyRadiomics (https://github.com/ Radiomics/PyRadiomics) to obtain radiomic features that adhere to the Imaging Biomarker Standardization Initiative [26]. PyRadiomics provides advanced computational capabilities for image analysis, enabling the extraction of a multitude of features from images through sophisticated processing and filtering techniques. The ResNet 101 algorithm was used for depth feature extraction. A detailed description of deep neural network to extract depth features in the Additional file 1: S5. To address concerns regarding overfitting owing to the high dimensionality of available features, a random split in a 7:3 training and validation dataset ratio was performed. The training cohort in this study comprised 73 patients, whereas the validation cohort had 32 patients. Feature selection and model construction were performed according to the training cohort.

### Feature selection and construction of five predictive models

**Fig. 1:**
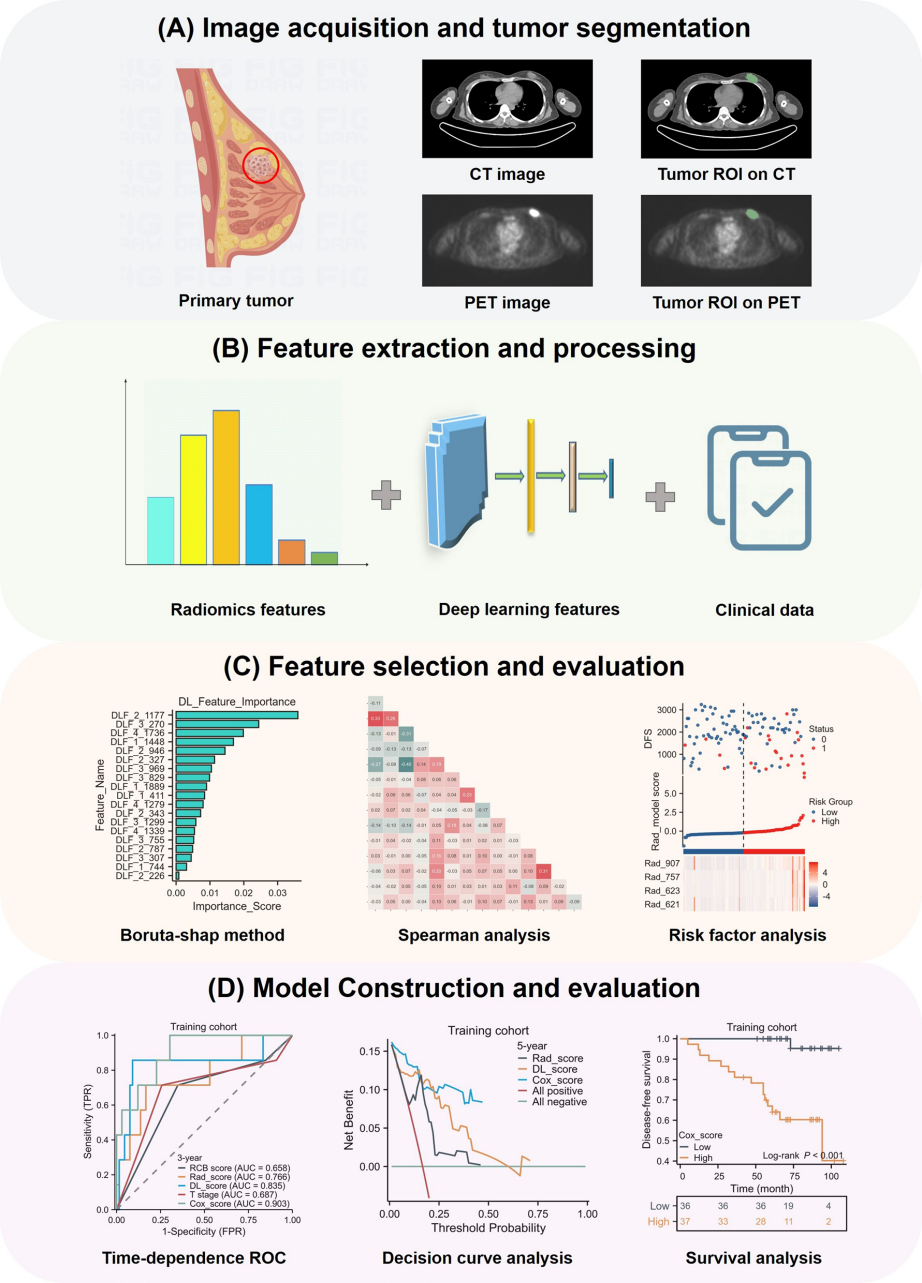
Outline of the workflow from tumor segmentation, feature extraction, selection, and model construction, evaluation

To ensure feature stability within the ROI, we randomly selected 30 patients and conducted two separate ROI segmentation procedures performed by different radiologists. Then interclass correlation coefficient (ICC) of each feature was calculated. We employed the U test to select features exhibiting significant distinctions with prognosis to identify prognosis-correlated features. The Boruta method was used to calculate each feature’s Shapley value and the max shadow value. When a Shapley value was higher than the max shadow value, the corresponding feature was selected for further analysis. Finally, we used univariate analysis and the multivariate Cox analysis with multiple comparison correction to reduce the amounts of features. Then we used the final feature sets to construct models. After multivariate Cox analysis, 2 clinical, 4 radiomic, and 7 depth features were retained. Clinical features were retained to build two clinical models for predicting prognosis. Radiomic and deep prognosis models were built by using radiomic and depth features respectively. A combined model incorporating all available features was ultimately developed to predict prognosis. The procedure is summarized in Fig. 1.

### Statistical analysis

The Mann–Whitney U test was used to compare their inter-group difference. Univariate and multivariate analysis was conducted using the Cox proportional risk model to estimate the association between selected features and DFS. Hazard ratios (HR) and 95% confidence interval (CI) of each variable were obtained. A *p* < 0.05 was considered statistically significant. Detailed information is presented in Additional file 1: Table S1. We used time-dependent receiver operating characteristic (ROC) curves and the area under the ROC curve (AUC) to evaluate and compare the predictive performance of different models. Decision curve analysis (DCA) was employed to assess the strength and clinical relevance of the models.

## Results

### Patient and tumor characteristics

This retrospective study analyzed data from 105 female non-pCR breast cancer patients who underwent NAC, with 73 patients allocated to the training group and 32 patients assigned to the validation group. Patients were diagnosed at a mean age of 47 years and followed up for a median of 71 months. Among the patient population, 47 were diagnosed with clinical stage II disease, while 58 were diagnosed with clinical III stage disease. At the time of the last follow-up, 15 disease relapses and 7 deaths occurred, while 83 patients were alive without disease recurrence. Clinicopathologic features, including molecular subtypes, RCB score, menstrual status, and SUV, among other parameters, are presented in Table 1.

**Table 1:** Baseline characteristics of patients

Baseline characteristics of the study population population (*n* = 105)	*N* (%)
*Age, years*	
Mean	47
SUVmean	3.92 ± 2.05
SUVmax	6.25 ± 3.73
SUVmin	1.63 ± 0.69
*ER status*	
Positive	83 (79.05%)
Negative	22 (20.95%)
*PR status*	
Positive	88 (83.8%)
Negative	17 (16.2%)
*Ki67*	
Positive	83 (79.05%)
Negative	22 (20.95%)
*Molecular subtype*	
HR-positive/HER2-negative	66 (62.29%)
HER2-positive	31 (29.52%)
TNBC	8 (8.19%)
*Menopausal status*	
Premenopausal	37 (35.24%)
Postmenopausal	68 (64.76%)
*Prechemotherapy T stage*	
T1	12 (11.43%)
T2	56 (53.33%)
T3	19 (18.1%)
T4	18 (17.14%)
*Prechemotherapy N stage*	
N0	21 (20%)
N1	36 (34.29%)
N2	35 (33.33%)
N3	13 (12.38%)
*Prechemotherapy stage*	
II	47 (44.76%)
III	58 (55.24%)
*Event*	
Yes	22 (20.95%)
No	83 (79.05%)
*RCB*	
I	15 (14.29%)
II	48 (45.71%)
III	42 (40%)

*ER* estrogen receptor; *PR* progesterone receptor; *HER2* human epidermal growth factor 2; *HR* hormone receptor positive; *TNBC* triple-negative breast cancer; *RCB* residual cancer burden

### Feature extraction and selection for model construction

3644 radiomic features and 4096 depth features were extracted. To ensure the reproducibility of the features, a total of 1945 ineligible features with an ICC < 0.75 were excluded. The Mann–Whitney U test showed that 1536 features were significantly associated with prognosis. Then we performed independent Boruta feature selection. The Boruta method was used to select the important and robust features with higher Shapley values than the max shadow value by 1000 internals bootstrap. Finally, we used univariate analysis and the multivariate Cox analysis to reduce the amounts of features. We extracted and retained 4 radiomic features and 7 depth features. The results of univariate analysis and the multivariate Cox analysis with multiple comparison correction are presented Additional file 1: S3.

**Fig. 2:**
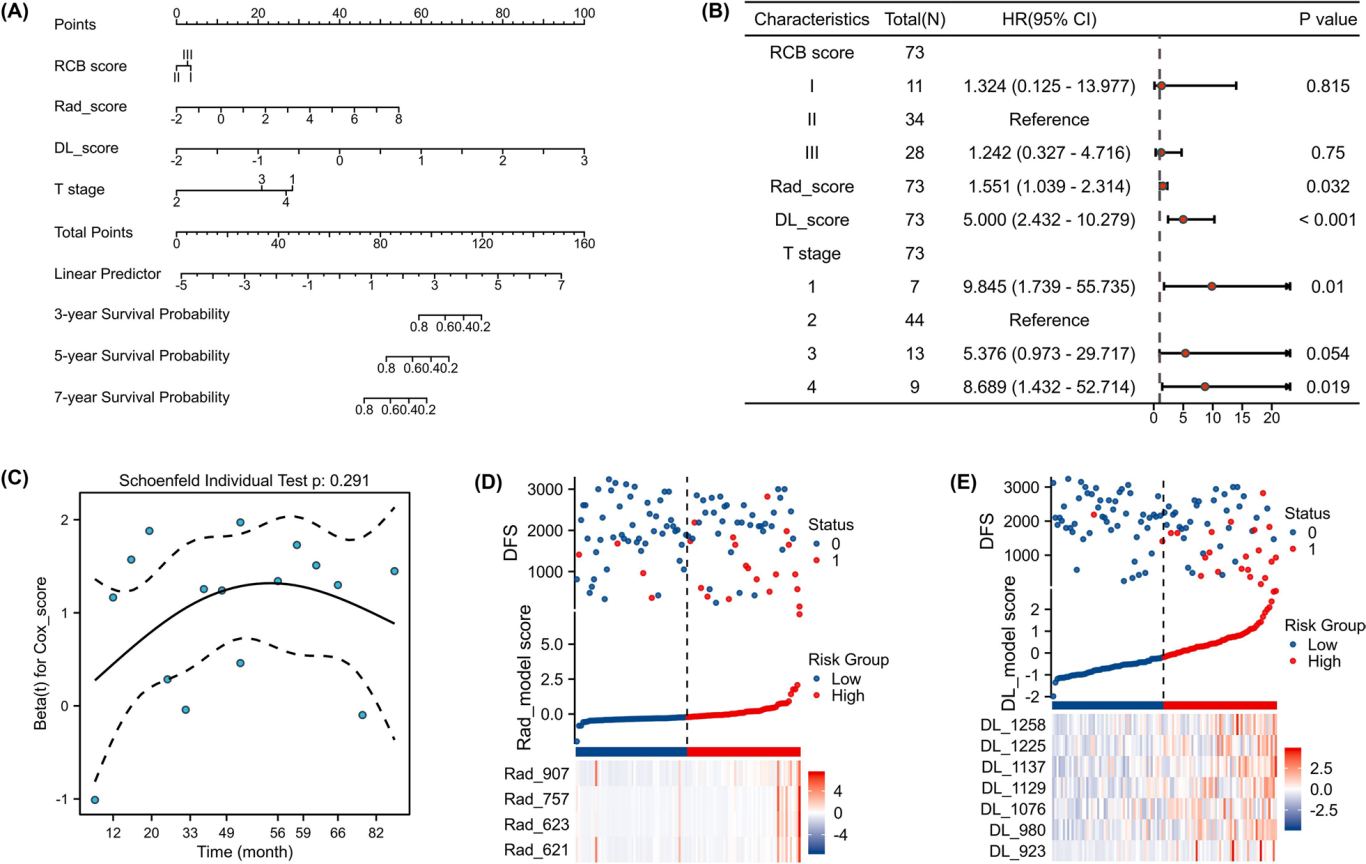
Feature selection and a nomogram of prediction model. **a** Schematic diagram of prediction model. Each factor corresponded to one point, the scores of the four factors were summed to give a total score, and finally the corresponding 3-year, 5-year and 7-years survival rates were calculated. **b** Multivariate analysis showed different independent prognostic factors. **c** Schoenfeld residual test. The selected features in the radiomics model (**d**), DL model (**e**)

The radiomic features constitute a diverse set of feature groups, encompassing a range of methodologies including first-order statistical analysis and shape-based analysis. Detailed descriptions of these features can be obtained from Additional file 1: Table S2. Based on Cox analysis method, 4 radiomic features (Fig. 2D) and 7 depth features (Fig. 2E) associated with prognosis were ultimately selected for building the radiomics and DL models. Two clinical features (cT and RCB) were retained to construct clinical models. A combined model was also developed, utilizing clinical scores, radiomic scores, and depth scores to prognosticate non-PCR breast cancer patients after NAC. In Fig. 2A, the survival rates after 3 years and 5 years are shown. The name of the final retained radiomic features is presented in Additional file 1: S6.

### Predictive performance of the five models

**Fig. 3:**
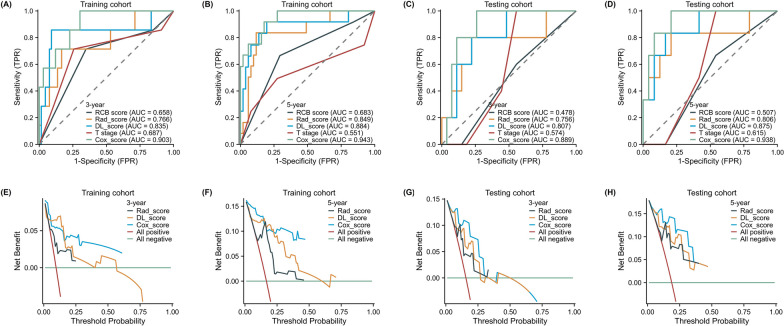
Training and testing cohort of ROC curve and DCA curve. The receiver operating characteristic (ROC) curves of the radiomics model, DL model, clinical models (T, RCB) and combined model in the training cohort (**a**, **b**) and validation cohort (**c**, **d**). The combined model demonstrated significantly higher AUCs in the training and validation cohorts than other models. Decision curve analysis (DCA) of the radiomics model, DL model and combined model in the training cohort (**e**, **f**) and validation cohort (**g**, **h**). The x-axis is the threshold probability, and the y-axis measures the net benefit. The combined model received a higher net benefit than the other two models across most ranges of reasonable threshold probabilities

**Fig. 4:**
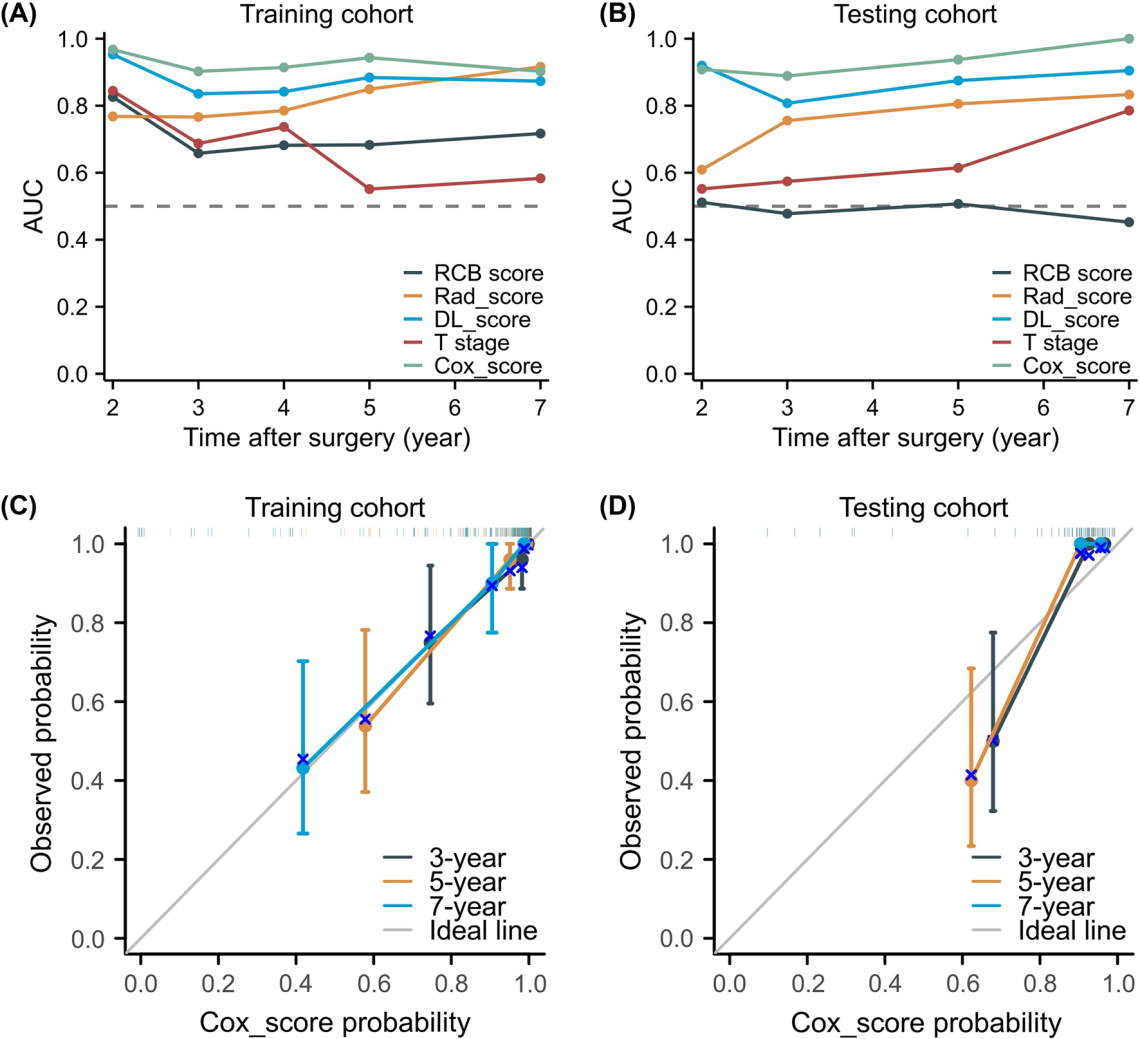
Predictive performance of the five models in training cohort (**a**) and test cohort (**b**). Training cohort (**c**) and validation cohort **d** calibration curve

The combined model demonstrated the highest predictive ability for breast cancer prognosis in the training cohort, with an AUC value of 0.903 and 0.943 for 3-year and 5-year survival, respectively. AUC values of 0.835 and 0.884 were predicted by the DL model for 3-year and 5-year survival, while the radiomics model performed slightly lower, with AUC values of 0.766 and 0.849 for 3-year and 5-year survival, respectively. In contrast, the clinical models established using cT and RCB had poor predictive efficacy, with 5-year AUC values of 0.683 and 0.551, respectively. In the validation cohort, the combined model outperformed the other models demonstrating an AUC value of 0.889 and 0.938 for 3-year and 5-year survival, respectively. The deep learning and radiomics models showed AUC values of 0.875 and 0.806 for 5-year survival, respectively, while the clinical models had low predictive power with 5-year AUC values of 0.615 and 0.507 for cT and RCB, respectively. These outcomes indicate that the combined model is a more accurate predictor of DFS for non-pCR patients after NAC than other models. Figure 3 provides a comprehensive comparison of the five models, and ROC analysis and DCA curves confirmed the superiority of the combined model over other models in both the training and validation groups. Figure 4 shows predictive performance of the five models in training cohort and test cohort.

### Stratification of disease-free survival by the five predictive models

**Fig. 5:**
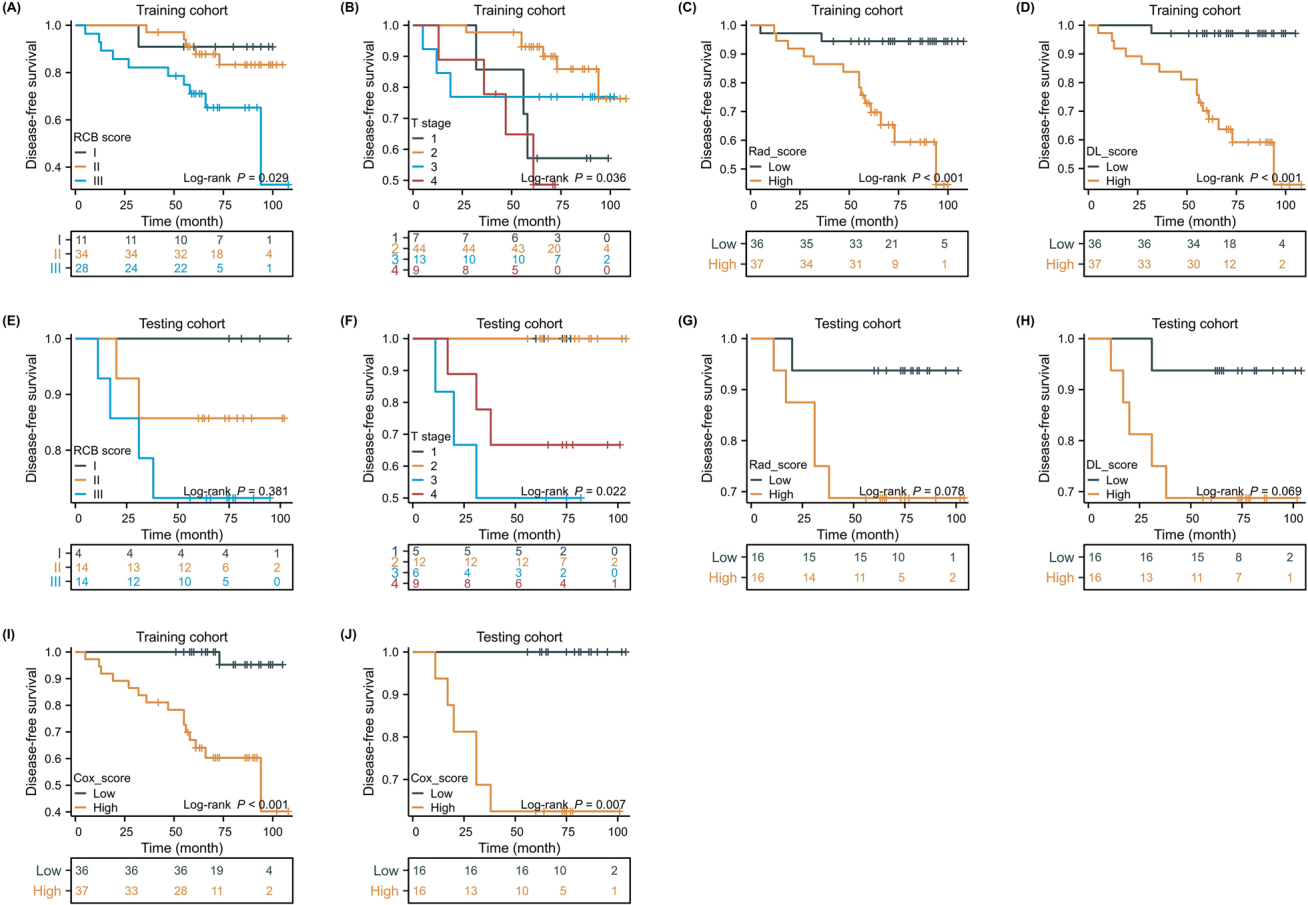
Disease-free survival (DFS) curves of patients stratified by RCB model (**a**), clinical T model (**b**), radiomics model (**c**), DL model (**d**) and combined model (**i**) in the training cohort. The DFS curves of patients stratified by RCB model (**e**), clinical T model (**f**), radiomics model (**g**), DL model (**h**) and combined model **j** in the testing cohort. The DFS showed a significant difference between patients in combined model in training and testing cohort. Among all models, patients stratified by a combined model demonstrated the best capability in stratifying patients with high risk

All models were used to stratify the predicted survival of non-pCR patients after NAC. In the training cohort, DFS exhibited significant differences among breast cancer patients stratified by clinical, radiomic, DL, and combined models. The combined model demonstrated continued satisfactory stratification in the validation set, whereas the DL and radiomics models showed slightly poorer stratification. The integrated model had the best stratification ability among all models in identifying high-risk patients (Fig. 5).

## Discussion

Our study introduced and validated an integrated model that accurately stratifies non-pCR breast cancer patients based on clinicopathologic features, depth, and radiomic features extracted from PET/CT images obtained before NAC administration. The integrated model secured the highest AUC values among the independent validation cohort with a 3-year survival AUC value reaching 0.889 and a 5-year survival AUC value of 0.938, respectively.

We found that the model combining radiomic, depth features and clinicopathologic factors achieved better predictive performance than individual prognostic factors. Though some studies showed that patients with an increased RCB score have a high risk of a worse prognosis and shorter survival time [9, 27], and others have indicated that combining RCB and KI67 can provide better predictions than the RCB system [10, 28], highlighting the importance of including more comprehensive and meaningful information in prediction models. In both the training set and validation set, our combined model consistently outperforms the RCB model alone in accurately predicting the prognosis of breast cancer patients, with an AUC value of 0.943 and 0.938 for 5-year survival, respectively. This underscores the added value and potential synergistic effect of integrating radiomics with traditional clinical and pathological information for more accurate prognostic predictions in breast cancer patients.

Individual metabolic factors were not a reliable predictor of survival. PET/CT allows for the simultaneous assessment of metabolic and structural functions, with research primarily focusing on PET/CT metabolic parameters such as standard uptake value (SUV) and metabolic tumor volume (MTV) in predicting the prognosis of breast cancer patients [29–33]. Nonetheless, semi-quantitative parameters obtained from PET/CT images have certain limitations in their capacity to fully capture the heterogeneity of breast cancer. For instance, while SUVmax denotes solely the hottest pixel, MTV is reliant upon methods that are based on thresholds. Our study also included metabolic indicators such as SUVmax as relevant clinical factors for prognostic analysis, but individual metabolic factors alone did not improve predictive performance. This finding supports the controversy surrounding the inconsistency between ^18^F-FDG tumor uptake and prognosis prediction, which might be due to tumor heterogeneity and different research methods [34, 35].

We successfully developed an integrated model based on depth and radiomic features from PET images that accurately predicted long-term survival in non-pCR breast cancer patients. Radiomics and deep learning are efficient diagnostic tools with a variety of clinical applications [36, 37]. The extraction of numerous image features from the region of interest is achieved through the utilization of mathematical algorithms in these approaches [38], and non-invasive biomarkers derived from PET radiomics can be generated based on a range of pixel intensities, associated parameters, and positions of the images. [39]. Several studies were exploring the clinical and technical viability of PET radiomics for breast cancer diagnosis [40, 41], staging [42, 43], pathological characterization [44, 45], and prediction of response to NAC [46–48]. Clément Bouron [49] investigated to evaluate the prognostic value of baseline PET/CT metabolic parameters, volumetric parameters, and texture parameters for early TNBC breast cancer. The study revealed that imaging feature entropy demonstrated potential as a prognostic indicator. David [50] discovered that texture features exhibited a significant correlation with OS and DFS in patients with advanced breast cancer. However, few studies have investigated PET/CT radiomics and depth features for prognosis prediction in non-pCR breast cancer patients. In this study, our combined model incorporating tumor stage, RCB, radiomic, and depth features exhibited excellent performance, with a five-year survival AUC of 0.943 and 0.938 in the training and validation cohorts. The combined model also demonstrated robust clinical usefulness with greater benefits in both the training and validation cohorts in DCA curve analysis.

The limitations of the present study include a single-center design and retrospective methodology. To establish the validity and generalizability of our findings, additional research is warranted. While our deep learning model performed better than the radiomics model in the training set, further investigation is necessary to elucidate the interpretability of feature sources associated with this approach.

## Conclusion

In conclusion, we formulated a comprehensive model that integrates radiomic and depth features obtained from PET/CT scans to forecast DFS in non-pCR patients. This amalgamated model serves as an efficient approach for projecting DFS in non-pCR patients diagnosed with breast cancer following NAC, potentially facilitating treatment refinement for individuals at high risk and thereby enhancing overall survival.

### Supplementary Information

**Additional file 1**. **Table S1**. The results of cox analysis. **Table S2**. Summary of radiomics features. **Table S3**. Univariate and multivariate analyses with a Bonferroni calibration test. **Table S4**. Image pre-processing. **Table S5**. Basic principles of deep learning and neural networks. **Table S6**. The name of the final retained radiomic features.

## Data Availability

The datasets used and analyzed during the current study are available from the corresponding authors on reasonable request.

## Abbreviations

DFS: Disease-free survival
pCR: Pathological complete response
NAC: Neoadjuvant chemotherapy
OS: Overall survival
RCB: Residual cancer burden
NCCN: National Comprehensive Cancer Network
^18^F-FDG PET/CT: ^18^F-2-deoxy-2-fluoro-d-glucose (FDG) positron emission tomography/computed tomography
DL: Deep learning
Ki67: Proliferative activity
HER2: Human epidermal growth factor receptor 2
IHC: Immunohistochemical
FISH: Fluorescence in situ hybridization
3D-ROI: Three-dimensional area of interest
HR: Hazard ratios
CI: Confidence interval
ROC: Receiver operating characteristic
DCA: Decision curve analysis
SUV: Standard uptake value
MTV: Metabolic tumor volume
ER: Estrogen receptor
PR: Progesterone receptor
HR: Hormone receptor
TNBC: Triple-negative breast cancer
ICC: Interclass correlation coefficient
