# Supplementary material for: 18F-FDG PET/CT-based deep learning radiomics predicts 5-years disease-free survival after failure to achieve pathologic complete response to neoadjuvant chemotherapy in breast cancer
Source: EJNMMI Res. 2023 Dec 6;13:105. doi: 10.1186/s13550-023-01053-7 (PMC10697916; doi:10.1186/s13550-023-01053-7)
Supplement: Supplementary file 1 — Additional file 1. Table S1. The results of cox analysis. Table S2. Summary of radiomics features. Table S3. Univariate and multivariate analyses with a Bonferroni calibration test. Table S4. Image pre-processing. Table S5. Basic principles of deep learning and neural networks. Table S6. The name of the final retained radiomic features. [file 13550_2023_1053_MOESM1_ESM.docx]

**^18^F-FDG PET/CT-based Deep Learning Radiomics Predicts Five-years Disease-free Survival After Failure to Achieve Pathologic Complete Response to Neoadjuvant Chemotherapy in Breast Cancer**

Xingxing Zheng^1^ · Yuhong Huang^1^ · Yingyi Lin^2^ · Teng Zhu^1^ · Jiachen Zou^1,3^ · Shuxia Wang^4^ · Kun Wang^1^

**European Journal of Nuclear Medicine and Molecular Imaging Research**

^1^ Department of Breast Cancer, Cancer Center, Guangdong Provincial People's Hospital (Guangdong Academy of Medical Sciences), Southern Medical University, Guangzhou, China

^2^ Shantou University Medical College, Shantou, China

^3^ Guangdong Medical University, Zhanjiang, China

^4^ Department of Nuclear Medicine and PET centre, Guangdong Provincial People's Hospital (Guangdong Academy of Medical Sciences), Southern Medical University, Guangzhou, China

Correspondence

Kun Wang, Department of Breast Cancer, Cancer Center, Guangdong Provincial People's Hospital (Guangdong Academy of Medical Sciences), Southern Medical University, Guangzhou, China. Email: gzwangkun@126.com; ORCID: 0000-0001-9851-7080

Shuxia Wang, Department of Nuclear Medicine and PET centre, Guangdong Provincial People's Hospital (Guangdong Academy of Medical Sciences), Southern Medical University, Guangzhou, China. Email: wangshuxia@gdph.org.cn

Supplementary Table 1. The results of cox analysis

| Characteristics | Total(N) | Univariate analysis | |  | Multivariate analysis | |
| --- | --- | --- | --- | --- | --- | --- |
|  |  | Hazard ratio (95% CI) | P value |  | Hazard ratio (95% CI) | P value |
| RCB score | 73 |  | **0.037** |  |  |  |
| II | 34 | Reference |  |  | Reference |  |
| III | 28 | 3.296 (1.115 - 9.750) | **0.031** |  | 1.242 (0.327 - 4.716) | 0.750 |
| I | 11 | 0.611 (0.071 - 5.233) | 0.653 |  | 1.324 (0.125 - 13.977) | 0.815 |
| Rad_score | 73 | 1.980 (1.449 - 2.706) | **< 0.001** |  | 1.551 (1.039 - 2.314) | **0.032** |
| DL_score | 73 | 3.471 (2.198 - 5.481) | **< 0.001** |  | 5.000 (2.432 - 10.279) | **< 0.001** |
| SUVmean | 73 | 0.994 (0.783 - 1.260) | 0.959 |  |  |  |
| SUVmax | 73 | 1.000 (0.880 - 1.137) | 0.999 |  |  |  |
| SUVmin | 73 | 0.916 (0.425 - 1.972) | 0.822 |  |  |  |
| Age | 73 | 1.000 (0.947 - 1.056) | 0.988 |  |  |  |
| Menses | 73 |  | 0.646 |  |  |  |
| presence | 50 | Reference |  |  |  |  |
| none | 23 | 1.271 (0.462 - 3.499) | 0.642 |  |  |  |
| Histological grading | 73 |  | 0.246 |  |  |  |
| 1 | 4 | Reference |  |  |  |  |
| 3 | 26 | 92394519.6988 (0.000 - Inf) | 0.998 |  |  |  |
| 2 | 43 | 67016127.3171 (0.000 - Inf) | 0.998 |  |  |  |
| T stage | 73 |  | 0.077 |  |  |  |
| 2 | 44 | Reference |  |  | Reference |  |
| 3 | 13 | 1.905 (0.475 - 7.635) | 0.363 |  | 5.376 (0.973 - 29.717) | 0.054 |
| 1 | 7 | 3.565 (0.890 - 14.282) | 0.073 |  | 9.845 (1.739 - 55.735) | **0.010** |
| 4 | 9 | 5.283 (1.441 - 19.370) | **0.012** |  | 8.689 (1.432 - 52.714) | **0.019** |
| N stage | 73 |  | 0.117 |  |  |  |
| 2 | 25 | Reference |  |  |  |  |
| 1 | 28 | 0.886 (0.285 - 2.754) | 0.835 |  |  |  |
| 0 | 14 | 0.266 (0.032 - 2.208) | 0.220 |  |  |  |
| 3 | 6 | 3.411 (0.838 - 13.888) | 0.087 |  |  |  |
| ER status | 73 |  | 0.837 |  |  |  |
| positive | 58 | Reference |  |  |  |  |
| negative | 15 | 1.144 (0.323 - 4.059) | 0.835 |  |  |  |
| PR status | 73 |  | 0.580 |  |  |  |
| positive | 64 | Reference |  |  |  |  |
| negative | 9 | 0.588 (0.077 - 4.477) | 0.608 |  |  |  |
| Ki-67 | 73 |  | 0.953 |  |  |  |
| negative | 14 | Reference |  |  |  |  |
| positive | 59 | 1.038 (0.295 - 3.646) | 0.954 |  |  |  |
| HER2+ | 73 |  | 0.714 |  |  |  |
| negative | 51 | Reference |  |  |  |  |
| positive | 22 | 0.812 (0.262 - 2.520) | 0.719 |  |  |  |
| Molecular subtype | 73 |  | 0.397 |  |  |  |
| HR+/HER2- | 47 | Reference |  |  |  |  |
| HER2+ | 22 | 0.756 (0.244 - 2.345) | 0.628 |  |  |  |
| TN | 4 | 0.000 (0.000 - Inf) | 0.998 |  |  |  |

Supplementary Table 2. Summary of radiomics features

| Feature classes | Feature names |
| --- | --- |
| Shape-based features  (n=14) | Voxel Volume, Mesh Volume, Maximum 3D diameter, Surface Area, Surface Area to Volume ratio, Sphericity, Major Axis Length, Spherical Disproportion, Maximum 2D diameter (Slice), Maximum 2D diameter (Column), Maximum 2D diameter (Row), Minor Axis Length, Least Axis Length, Elongation, Flatness |
| Grey level run length matrix (GLRLM) features  (n=16) | Short Run Emphasis (SRE), Long Run Emphasis (LRE), Grey Level Non-Uniformity (GLN), Grey Level Non-Uniformity Normalized (GLNN), Run Length Non-Uniformity (RLN), Run Length Non-Uniformity Normalized (RLNN), Run Percentage (RP), Grey Level Variance (GLV), Run Variance (RV), Run Entropy (RE), Low Grey Level Run Emphasis (LGLRE), High Grey Level Run Emphasis (HGLRE), Short Run Low Grey Level Emphasis (SRLGLE), Short Run High Grey Level Emphasis (SRHGLE), Long Run Low Grey Level Emphasis (LRLGLE), Long Run High Grey Level Emphasis (LRHGLE) |
| First order statistics features  (n=18) | Energy, Total Energy, Entropy, Minimum, 10th percentile, 90th percentile, Maximum, Mean, Median, Interquartile Range, Range, Mean Absolute Deviation (MAD), Robust Mean Absolute Deviation (rMAD), Root Mean Squared (RMS), Skewness, Kurtosis, Variance, Uniformity |
| Grey level dependence matrix (GLDM) features  (n=14) | Small Dependence Emphasis (SDE), Large Dependence Emphasis (LDE), Grey Level Non-Uniformity (GLN), Dependence Non-Uniformity (DN), Dependence Non-Uniformity Normalized (DNN), Grey Level Variance (GLV), Dependence Variance (DV), Dependence Entropy (DE), Low Grey Level Emphasis (LGLE), High Grey Level Emphasis (HGLE), Small Dependence Low Grey Level Emphasis (SDLGLE), Small Dependence High Grey Level Emphasis (SDHGLE), Large Dependence Low Grey Level Emphasis (LDLGLE), Large Dependence High Grey Level Emphasis (LDHGLE) |
| Grey level size zone matrix (GLSZM) features  (n=16) | Small Area Emphasis (SAE), Large Area Emphasis (LAE), Grey Level Non-Uniformity (GLN), Grey Level Non-Uniformity Normalized (GLNN), Size-Zone Non-Uniformity (SZN), Size-Zone Non-Uniformity Normalized (SZNN), Zone Percentage (ZP), Grey Level Variance (GLV), Zone Variance (ZV), Zone Entropy (ZE), Low Grey Level Zone Emphasis (LGLZE), High Grey Level Zone Emphasis (HGLZE), Small Area Low Grey Level Emphasis (SALGLE), Small Area High Grey Level Emphasis (SAHGLE), Large Area Low Grey Level Emphasis (LALGLE), Large Area High Grey Level Emphasis (LAHGLE) |
| Neighboring grey tone difference matrix (NGTDM) features  (n=5) | Coarseness, Contrast, Busyness, Complexity, Strength |
| Grey level co-occurrence matrix (GLCM) features  (n=24) | Autocorrelation, Joint Average, Cluster Prominence, Cluster Shade, Cluster Tendency, Contrast, Correlation, Difference Average, Difference Entropy, Difference Variance, Joint Energy, Joint Entropy, Informational Measure of Correlation (IMC) 1, Informational Measure of Correlation (IMC) 2, Inverse Difference Moment (IDM), Maximal Correlation Coefficient (MCC), Inverse Difference Moment Normalized (IDMN), Inverse Difference (ID), Inverse Difference Normalized (IDN), Inverse Variance, Maximum Probability, Sum Average, Sum Entropy, Sum of Squares |

|  | Characteristics | Total(N) | HR(95% CI) Univariate analysis | P value Univariate analysis | HR(95% CI) Multivariate analysis | adjust_p |  |
| --- | --- | --- | --- | --- | --- | --- | --- |
|  | F_621 | 73 | 1.000 (1.000 - 1.000) | < 0.01 | 1.000 (1.000 - 1.000) | < 0.01 |  |
|  | F_623 | 73 | 1.000 (1.000 - 1.000) | < 0.01 | 1.000 (1.000 - 1.001) | < 0.01 |  |
|  | F_757 | 73 | 1.000 (1.000 - 1.000) | < 0.01 | 1.000 (1.000 - 1.000) | < 0.01 |  |
|  | F_907 | 73 | 1.000 (1.000 - 1.000) | < 0.05 | 1.000 (1.000 - 1.000) | < 0.05 |  |
|  | F_923 | 73 | 1.000 (1.000 - 1.000) | < 0.05 | 1.000 (1.000 - 1.000) | < 0.05 |  |
|  | F_980 | 73 | 26.741 (3.740 - 191.171) | < 0.01 | 6.982 (0.541 - 90.161) | < 0.01 |  |
|  | F_1076 | 73 | 15.367 (1.232 - 191.634) | < 0.05 | 5.478 (0.385 - 77.901) | < 0.05 |  |
|  | F_1129 | 73 | 21.233 (3.119 - 144.525) | < 0.01 | 6.258 (0.780 - 50.191) | < 0.01 |  |
|  | F_1137 | 73 | 12.602 (2.893 - 54.883) | < 0.001 | 2.776 (0.408 - 18.907) | < 0.001 |  |
|  | F_1225 | 73 | 9.853 (1.993 - 48.724) | < 0.01 | 3.865 (0.633 - 23.593) | < 0.01 |  |
|  | F_1258 | 73 | 4.571 (1.306 - 15.998) | < 0.05 | 3.439 (0.555 - 21.315) | < 0.05 |  |

Supplementary material 3 Univariate and multivariate analyses with a Bonferroni calibration test

Supplementary material 4: image pre-processing

Before features extraction, the voxel size of each sequence was resampled to 1 × 1 × 1 mm3 and the bin width of gray-level histogram was fixed as 25. After z-score normalization of image pixel intensities, we extracted a total of 3644 quantitative imaging features. These encompassed shape-based features, first-order statistical features, textural features derived from original images, and other relevant features, employing corresponding Regions of Interest (ROIs).

Supplementary material 5: Basic principles of deep learning and neural networks

Deep learning is based on a deep neural network architecture and learns network weights for specific tasks from big data. The neural network is composed of many different types of layers, and the layer is also the basic unit of forward calculation. In this study, the two neural networks included convolutional layers, maximum pooling layers, average pooling layers, batch normalization layers, fully connected layers, activation function layers, and a global average pooling layer. The details of these layers are explained as follows:

(1) Convolutional layer. The convolutional layer adopts some filters (kernels) with a sliding stride and kernel size to calculate the weighted sum of the intensities of each point and its surrounding points of the input matrix. Its main function is to extract the features from the input matrix. For example, assuming an input matrix $I=\left( \begin{matrix} I_{11} & I_{12} & I_{13} \\ I_{21} & I_{22} & I_{23} \\ I_{31} & I_{32} & I_{33} \end{matrix} \right)$ and a filter $K=\left( \begin{matrix} k_{11} & k_{12} \\ k_{21} & k_{22} \end{matrix} \right)$ with a kernel size of 2 × 2 and a stride of 1, the output of the convolutional layer is

$F=conv(I, K)=\left( \begin{matrix} {I_{11}*k}_{11}+{I_{12}*k}_{12}{{+I}_{21}*k}_{21}+{I_{22}*k}_{22} & {I_{12}*k}_{11}+{I_{13}*k}_{12}{{+I}_{22}*k}_{21}+{I_{23}*k}_{22} \\ {I_{21}*k}_{11}+{I_{22}*k}_{12}{{+I}_{31}*k}_{21}+{I_{32}*k}_{22} & {I_{22}*k}_{11}+{I_{23}*k}_{12}{{+I}_{32}*k}_{21}+{I_{33}*k}_{22} \end{matrix} \right)$.

Output F is the channel of the feature map. By using multiple filters, a multi-channel feature map can be generated, which significantly improves the fitting ability of the convolutional layer. The neural network can extract more abstract features through continuous stacking of convolutional layers. In our study, the input images can be considered a two-dimensional (2D) matrix; thus, the 2D convolutional layers were used for feature extraction.

(2) Maximum pooling layer. The maximum pooling layer is mainly used to perform feature selection and dimensionality reduction on the input feature map. Effective feature selection and dimensionality reduction can improve robustness and reduce the complexity of the deep learning model. Assuming the feature map is $F=\left( \begin{aligned} \begin{matrix} 1 & 5 & \begin{matrix} 2 & 8 \end{matrix} \end{matrix} \\ \begin{matrix} 3 & 9 & \begin{matrix} 7 & 8 \end{matrix} \end{matrix} \\ \begin{matrix} 1 & 0 & \begin{matrix} 2 & 6 \end{matrix} \end{matrix} \\ \begin{matrix} 8 & 5 & \begin{matrix} 3 & 2 \end{matrix} \end{matrix} \end{aligned} \right)$, whose size is 4 × 4, the pooling sliding window is 2 × 2 with a stride of 2. The pooling operation divides matrix F into four disjoint sub-matrixes of size 2 × 2, and the maximum value of each small matrix is extracted to form the result matrix $P=\left( \begin{matrix} 9 & 8 \\ 8 & 6 \end{matrix} \right)$. In our study, one 2D max-pooling layer was used to perform feature selection and dimensionality reduction of images.

(3) Average pooling layer. The function of the average pooling layer is similar to that of the pooling layer. However, the calculation methods were different. For example, assuming that the feature map is also $F=\left( \begin{aligned} \begin{matrix} 1 & 5 & \begin{matrix} 2 & 8 \end{matrix} \end{matrix} \\ \begin{matrix} 3 & 9 & \begin{matrix} 7 & 8 \end{matrix} \end{matrix} \\ \begin{matrix} 1 & 0 & \begin{matrix} 2 & 6 \end{matrix} \end{matrix} \\ \begin{matrix} 8 & 5 & \begin{matrix} 3 & 2 \end{matrix} \end{matrix} \end{aligned} \right)$, the pooling sliding window is 2 × 2 with a stride of 2. Similar to the max-pooling layer, the average pooling operation divides the matrix F into four disjoint sub-matrixes of size 2 × 2. The average value, not the maximum value of each small matrix, will be calculated to generate the result matrix $P=\left( \begin{matrix} 4.5 & 6.25 \\ 3.5 & 3.25 \end{matrix} \right)$. In this study, an average pooling layer was used in each transition layer.

(4) Batch normalization layer. The batch normalization layer can normalize each channel of a batch of feature maps based on its mean and variance in the training process [1]. During the test, the statistical parameters learned from the training samples were used to normalize the test samples. Using the batch normalization layer can significantly accelerate network training and alleviate overfitting. In our model, batch normalization layers were used after or before all the convolutional layers.

(5) Fully connected (FC) layers. The fully connected layer can be described as a matrix multiplication plus one bias term, that is, $FC\left( x \right)=Wx+b$. The fully connected layer is usually used at the top of the network to provide a global receptive field for the network, in order to overcome the shortcomings of the convolutional layer that has only a limited receptive field. Through the fully connected layer, the feature map can be fused and compressed simultaneously and converted into the probability output of each category. In this study, three fully connected layers were used for a two-category classification task.

(6) Activation-function layer. The operations in the convolutional and fully connected layers can also be expressed in the form of matrix multiplication. Matrix multiplication is a linear operation, and it is often difficult to fit complex problems using only linear operations. To strengthen the nonlinear fitting ability of the neural network, an activation function layer needs to be used. In this study, we adopted a rectified linear unit (ReLU) function layer ($ReLU\left( x \right)=max\left( 0,x \right)$) after most convolutional layers and fully connected layers in our networks.

(7) Global average pooling (GAP) layer. The function of the GAP layer is to calculate the average intensity of all elements in each channel of a feature map, and finally output a feature vector whose length is the same as the number of channels. The use of a GAP layer can significantly reduce the number of weights in the network and effectively alleviate overfitting. In this study, we used the GAP layer between the last convolutional layer and the first fully connected layer.

Supplementary material 6: the name of the final retained radiomic features

Rad_621: First order statistics features: Entropy

Rad_623: First order statistics features: Variance

Rad_757: Grey level size zone matrix (GLSZM) features：Grey Level Non-Uniformity Normalized (GLNN)

Rad_907: Grey level dependence matrix (GLDM) features：Small Dependence Low Grey Level Emphasis (SDLGLE)
